# Supplementary material for: Somatic mutations impose an entropic upper bound on human lifespan
Source: NPJ Aging. 2026 Jun 25;12(1):116. doi: 10.1038/s41514-026-00421-6 (PMC13493869; doi:10.1038/s41514-026-00421-6)
Supplement: Supplementary file 1 [file 41514_2026_421_MOESM1_ESM.pdf]

Supplementary information

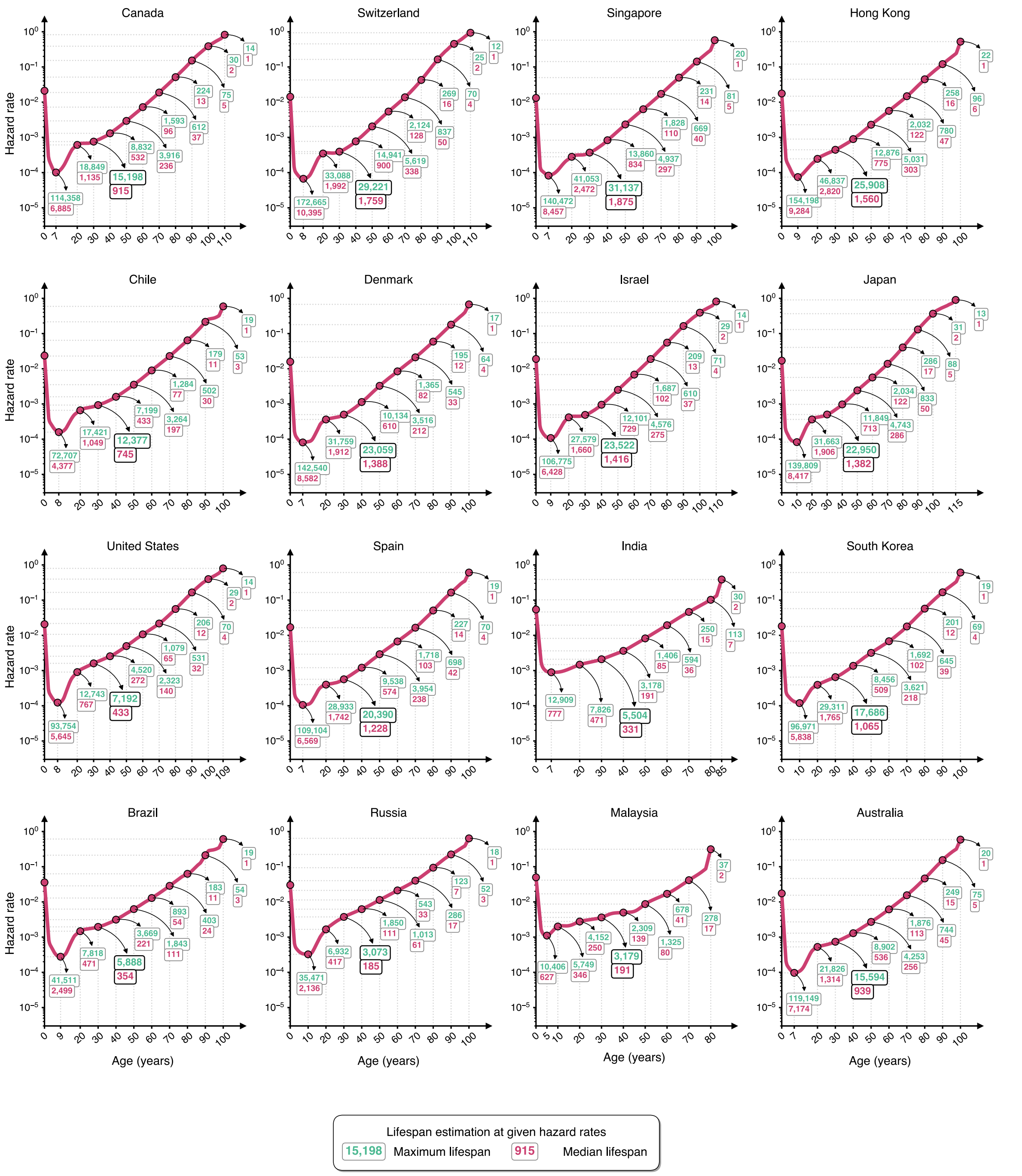

Supplementary Figure 1. Hazard rates and lifespans estimated for different ages in different populations. According to Model I, maximum and median lifespans are defined as ages at which the survival probability drops to 10^{-5} (or $10^{-3}\%$) and to 0.5 (or 50%), respectively. The lifespans calculated for the 30-year mortality are highlighted with black frames and larger font size.

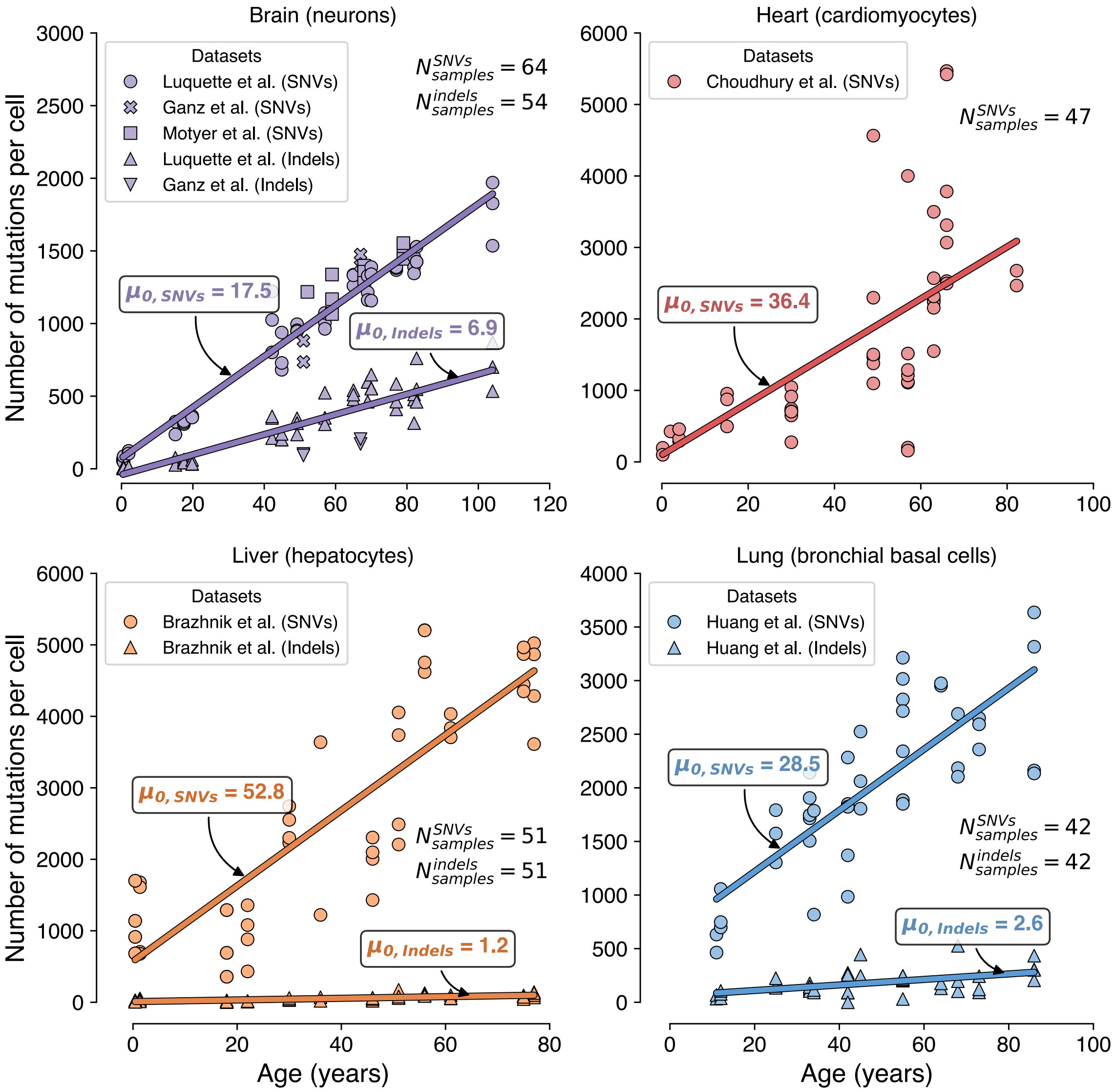

Supplementary Figure 2. Estimation of SNV and indel accumulation rates μ_0 across human lifespan in cell types included in the study.

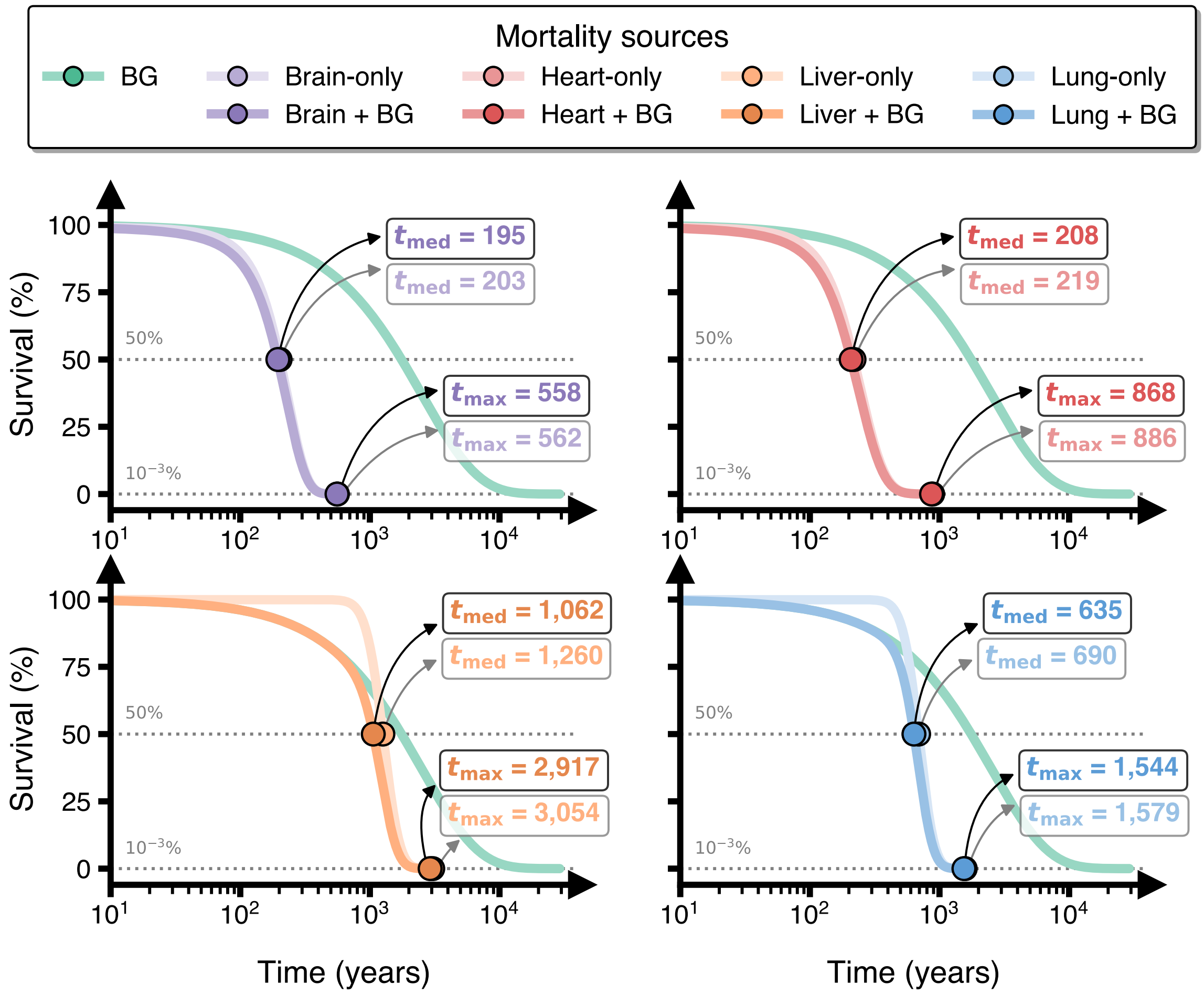

Supplementary Figure 3. Survival curves obtained by applying Model II for all organs included in the study. Horizontal dotted gray lines correspond to survival probabilities of 0.5 and 10^{-5}; median and maximum lifespans are annotated for organ-only and combined survival curves as t_{med} and t_{max}, respectively.

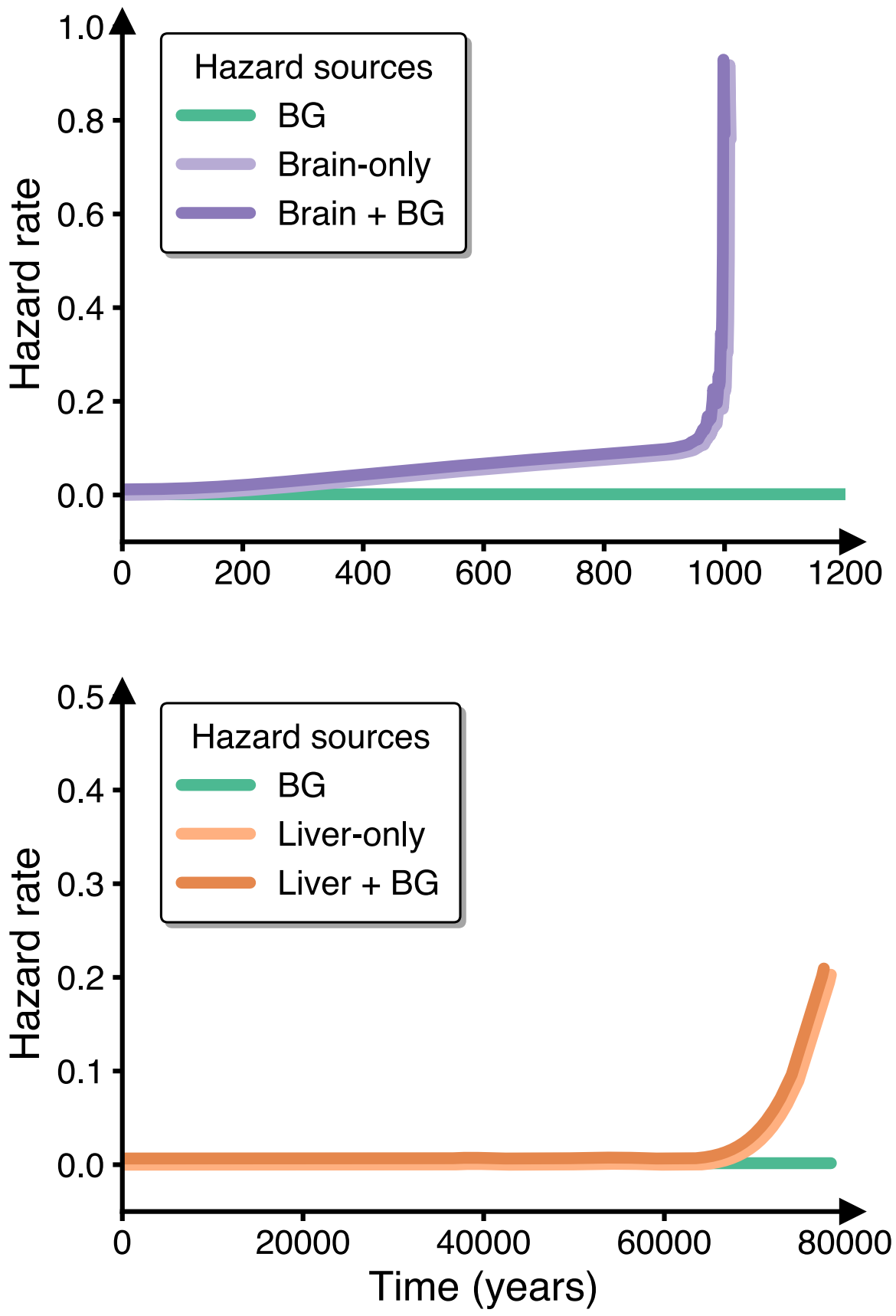

Supplementary Figure 4. Hazard rates obtained for Model II (brain and heart), Model IIIA (liver), and Model IIIC (lung). Model IIIB is not shown, because it yielded constant hazard rate. Organ-only and organ+BG curves overlap each other and are shown with a slight shift from each other for visualization purposes.

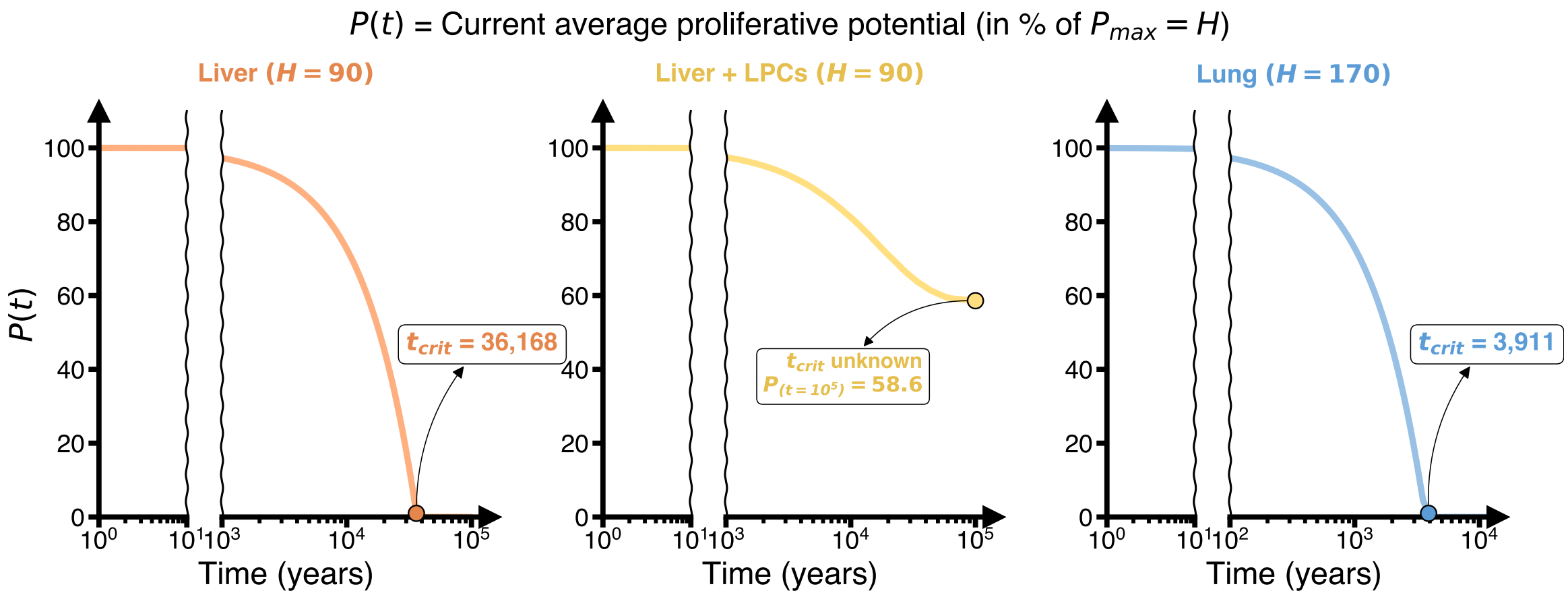

Supplementary Figure 5. Proliferative potential dynamics for Model IIIA (hepatocytes only), Model IIIB (hepatocytes in the presence of LPC population), and Model IIIC (lung BBCs). Shaded regions represent percentile bands (from the 2.5th to the 97.5th in 5% increments) for the simulated $P(t)$ trajectories; bright solid line corresponds to the median trajectory; the time at which the median potential trajectory crosses zero is annotated as t_{crit}.

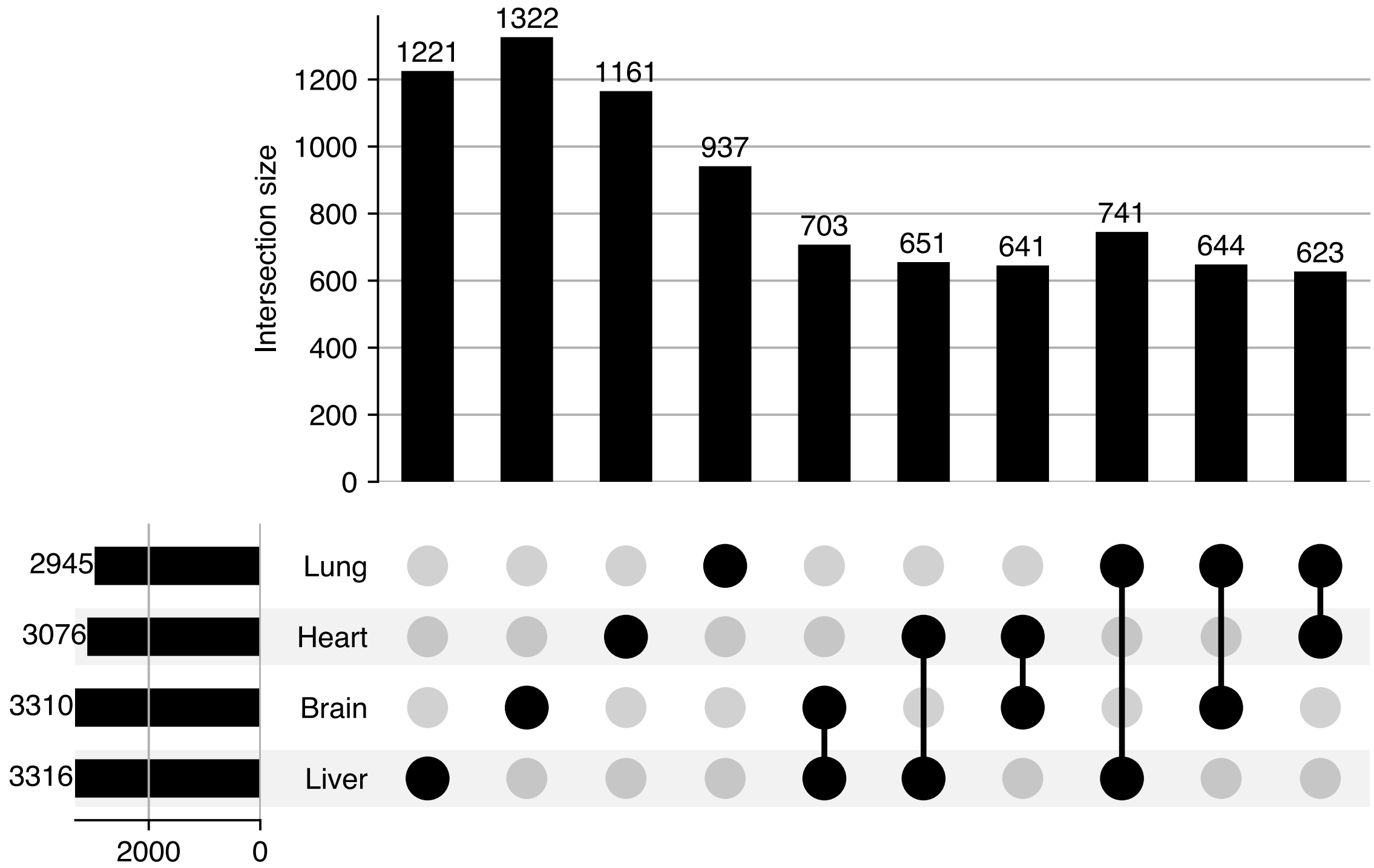

Supplementary Figure 6. Essential gene signature sizes and their overlaps inferred for each studied organ.

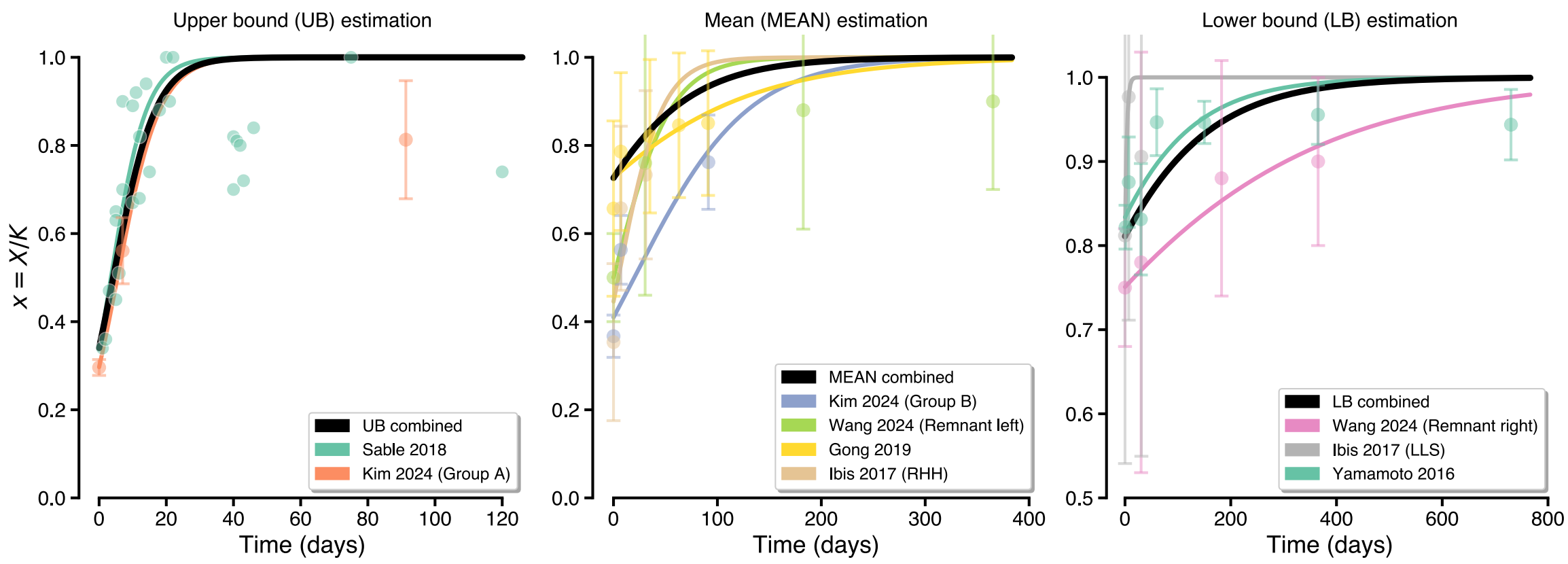

Supplementary Figure 7. Hepatocyte r estimation from studies on partial hepatectomy at different remnant liver volumes x. For the Sable 2018 dataset, points represent individual x measurements. For all other datasets, points represent median values aggregated over a 1-day time period, and error bars represent 95% CI. RHH, right hemi-hepatectomy; LLS, left lateral sectionectomy.

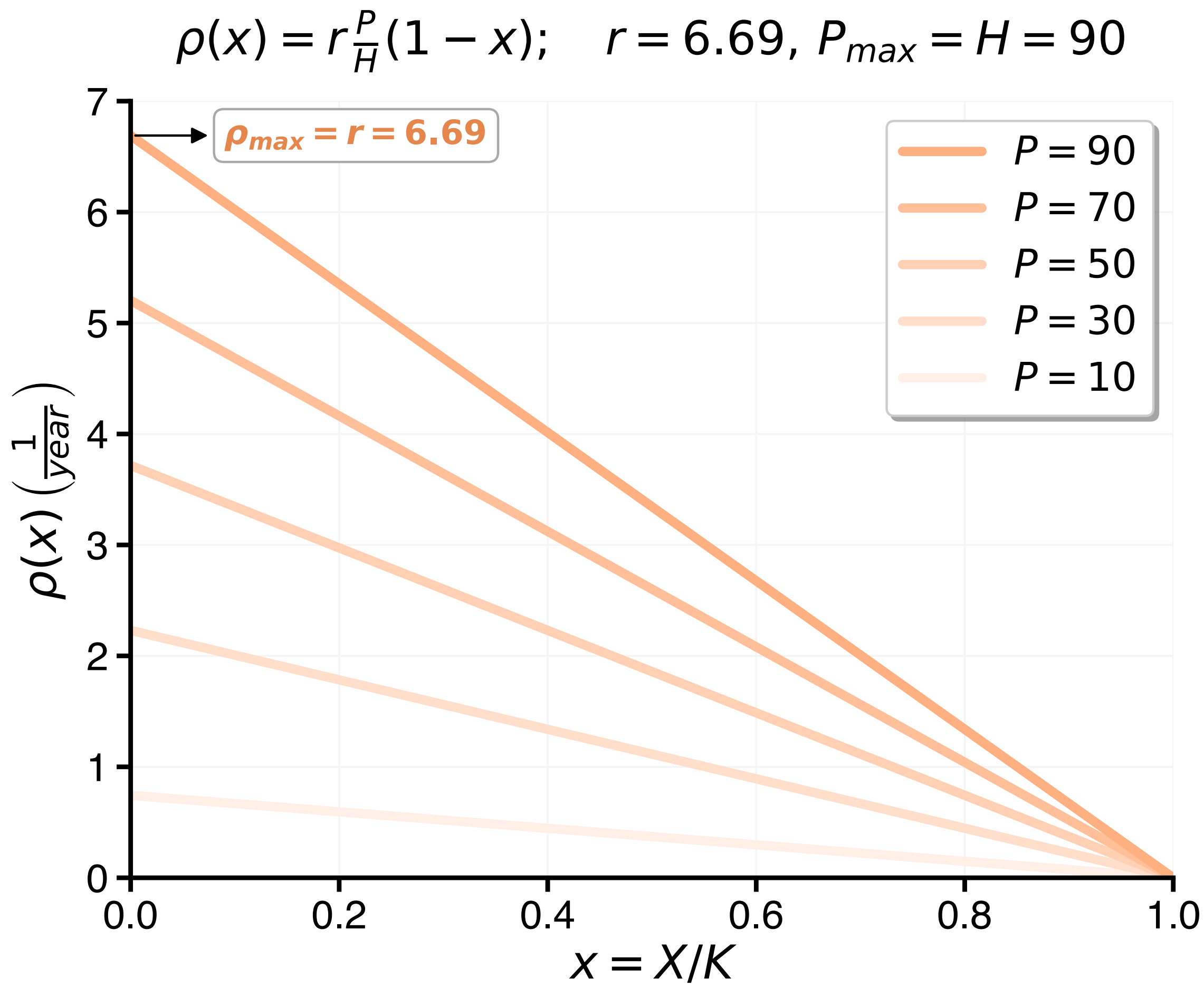

Supplementary Figure 8. Hepatocyte replication rate ρ as a function of both current hepatocyte population $x = X/K$ and average remaining proliferative potential P.

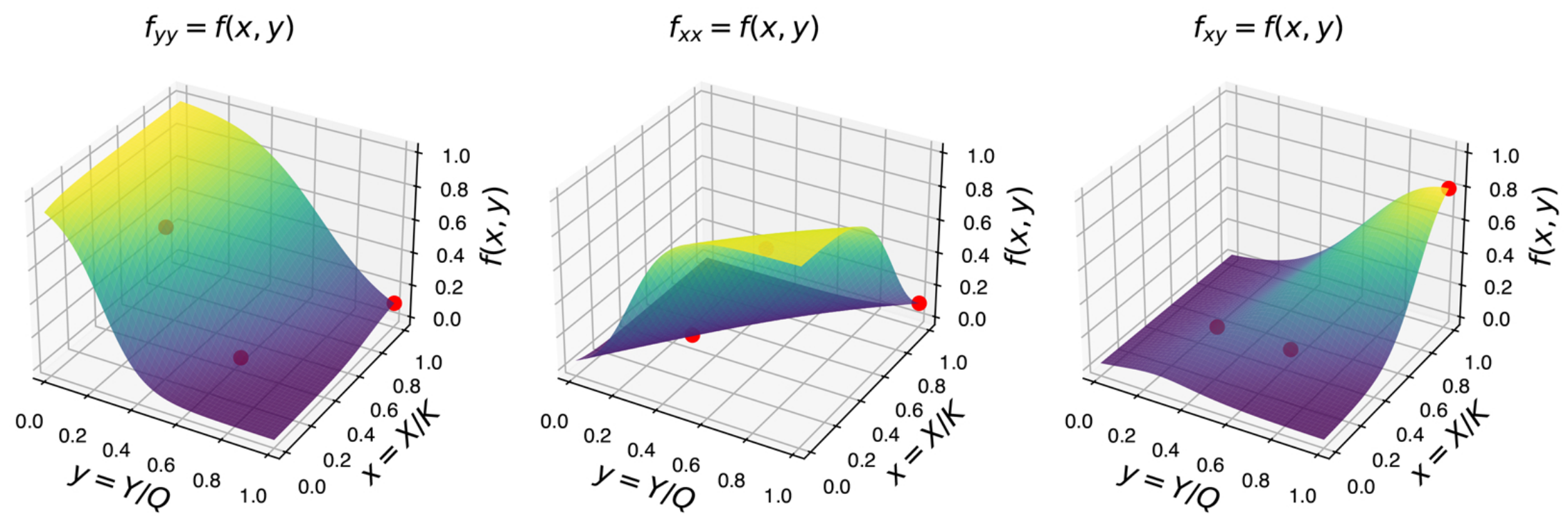

Supplementary Figure 9. Probability landscapes of liver progenitor cell (LPC) division outcomes as functions of somatic cell ($x = X/K$) and LPC ($y = Y/Q$) population ratios. Each surface $f_{...} = f(x,y)$ represents the probability that a stem cell undergoes symmetric self-renewal (f_{yy}, left), symmetric differentiation (f_{xx}, middle), or asymmetric division (f_{xy}, right), with red markers indicating reference points selected for function fitting.

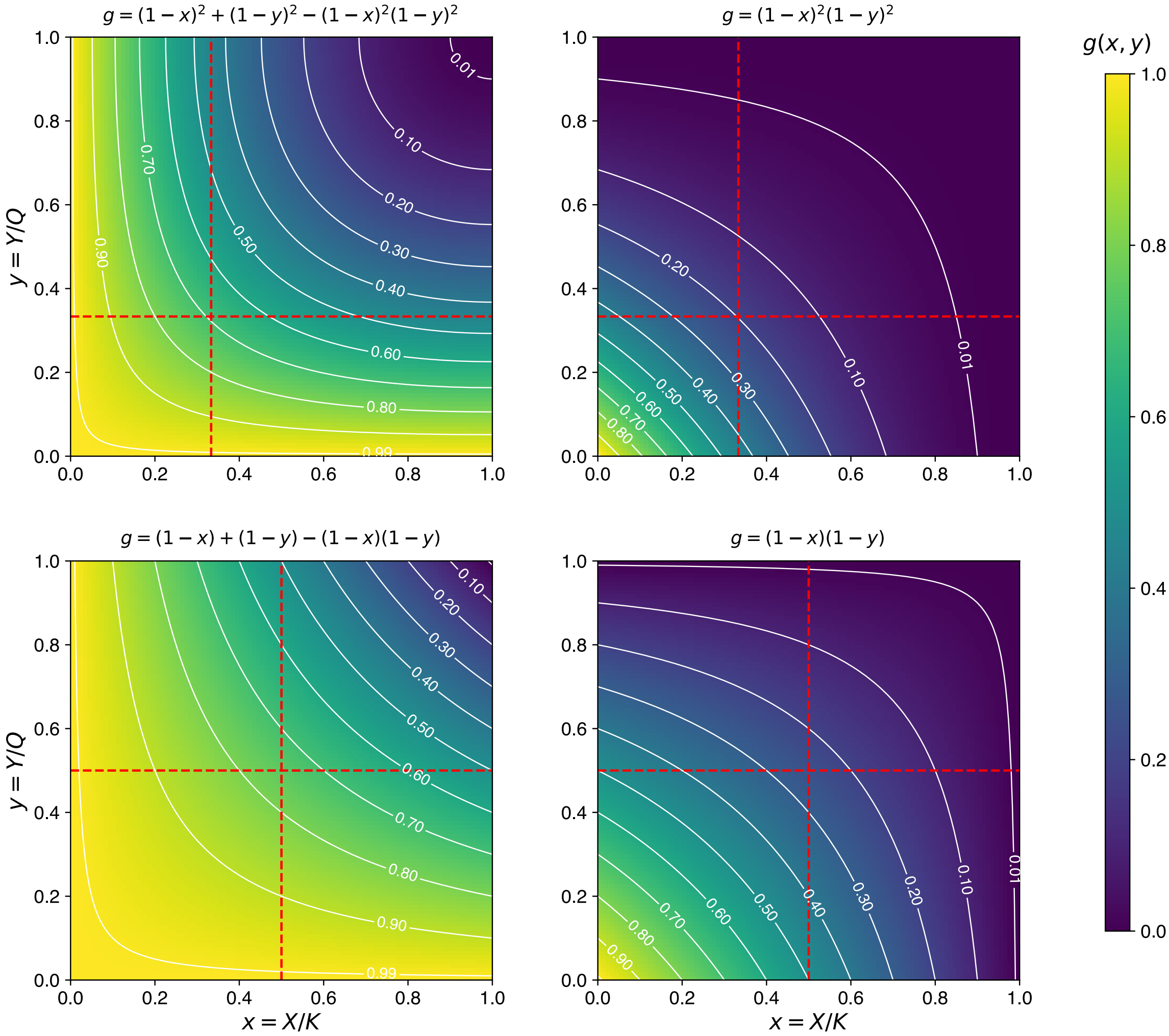

Supplementary Figure 10. Different candidate gate functions g for stem cell renewal rate. Each gate accounts both for somatic x and stem cell y populations at a given time. Color bar corresponds to different g values. White countour lines represent specific g values from 0.01 to 0.99 in 0.1 increments. Dashed red lines represent x and y values at inflection points 1/3 (top left and top right) and 1/2 (bottom left and bottom right).

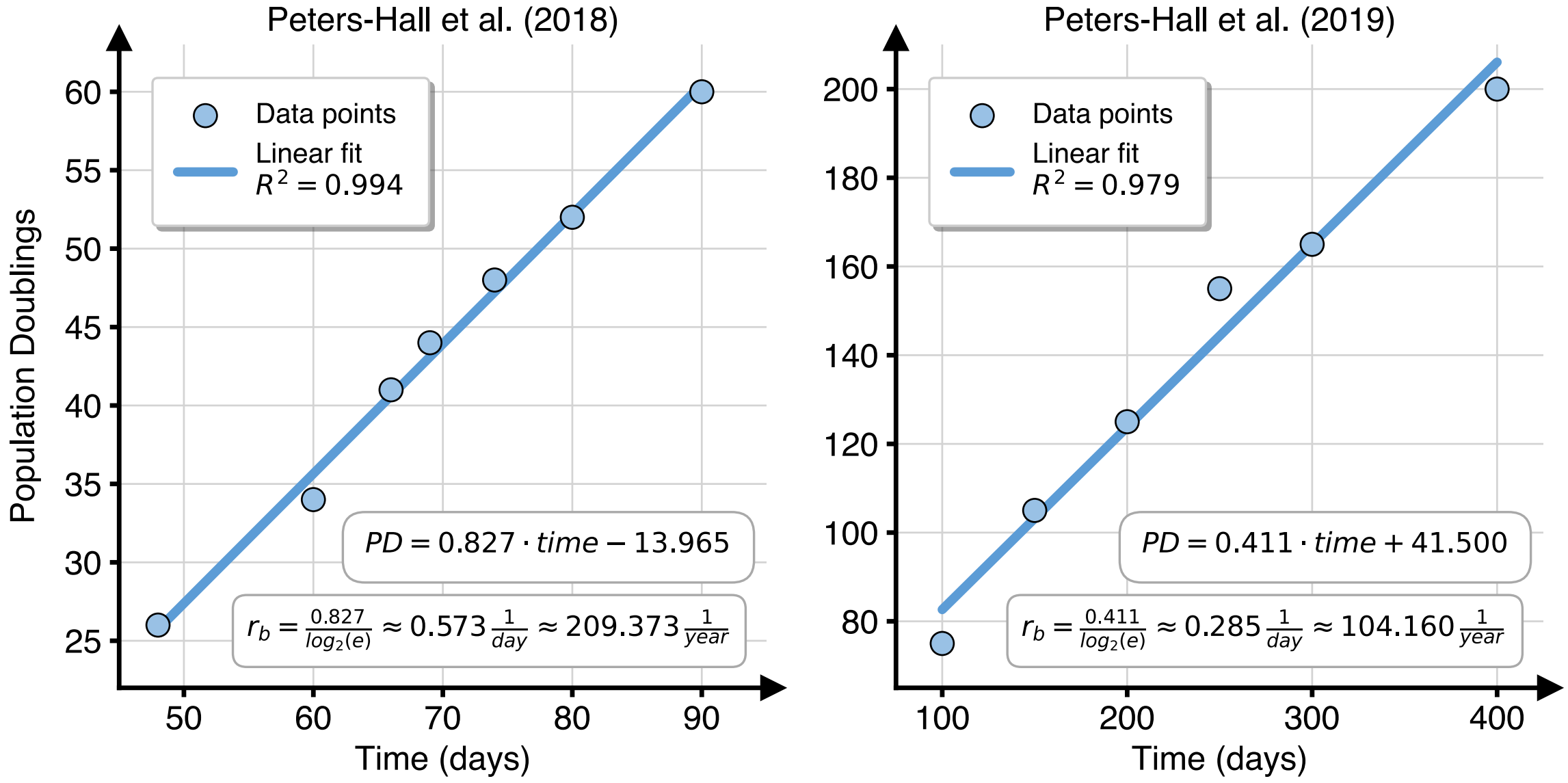

Supplementary Figure 11. Bronchial basal cells (BBC) r_b estimation from studies on *in vitro* BBC expansion. Only the period of linear growth is shown. PD, population doublings.

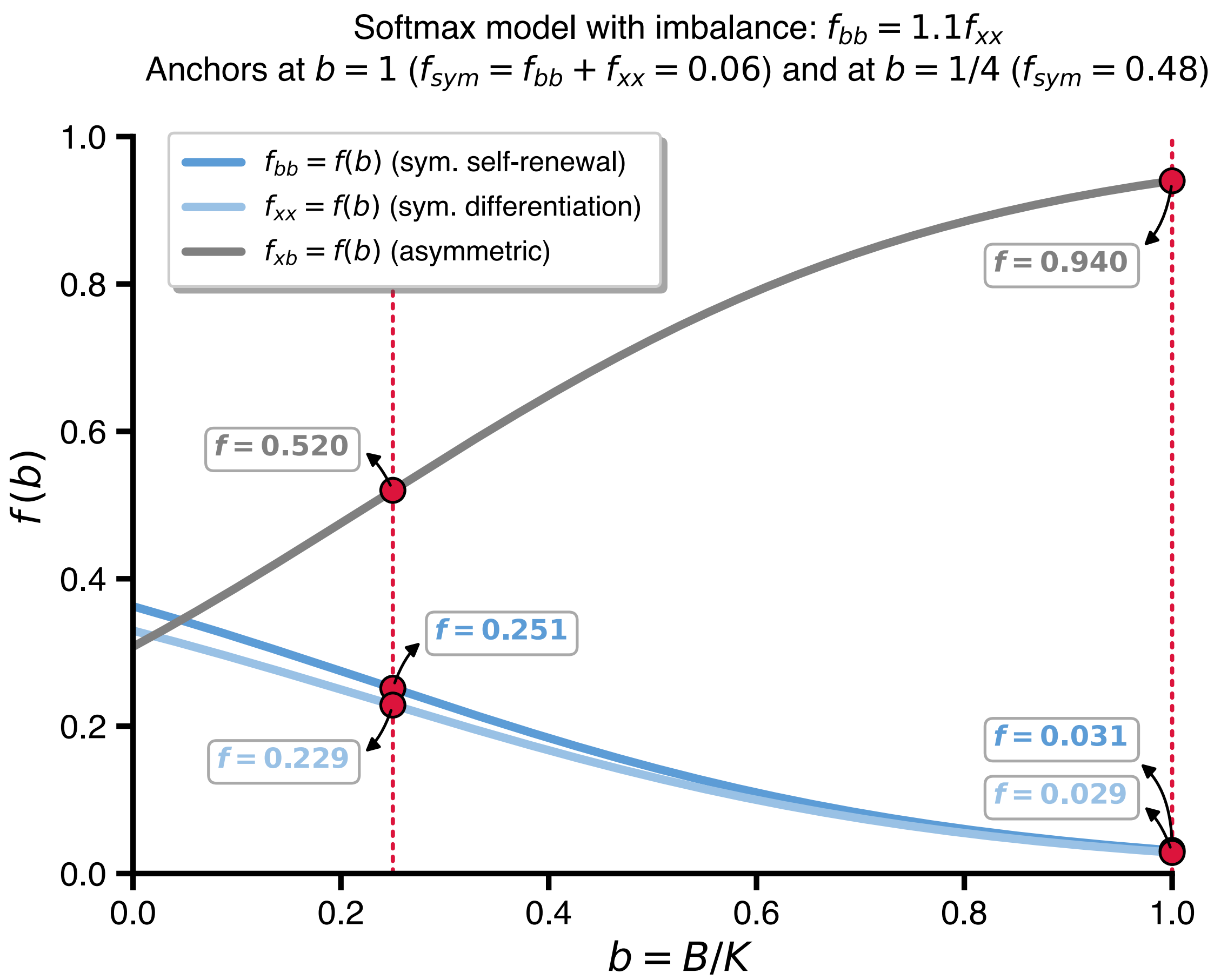

Supplementary Figure 12. Probability functions of different BBC division outcomes as functions of BBC population ratio $b = B/K$. Each $f(b)$ line represents the probability that a basal cell undergoes symmetric self-renewal (f_{bb}), symmetric differentiation (f_{xx}), or asymmetric division (f_{xb}), with red markers indicating reference points selected for function fitting.

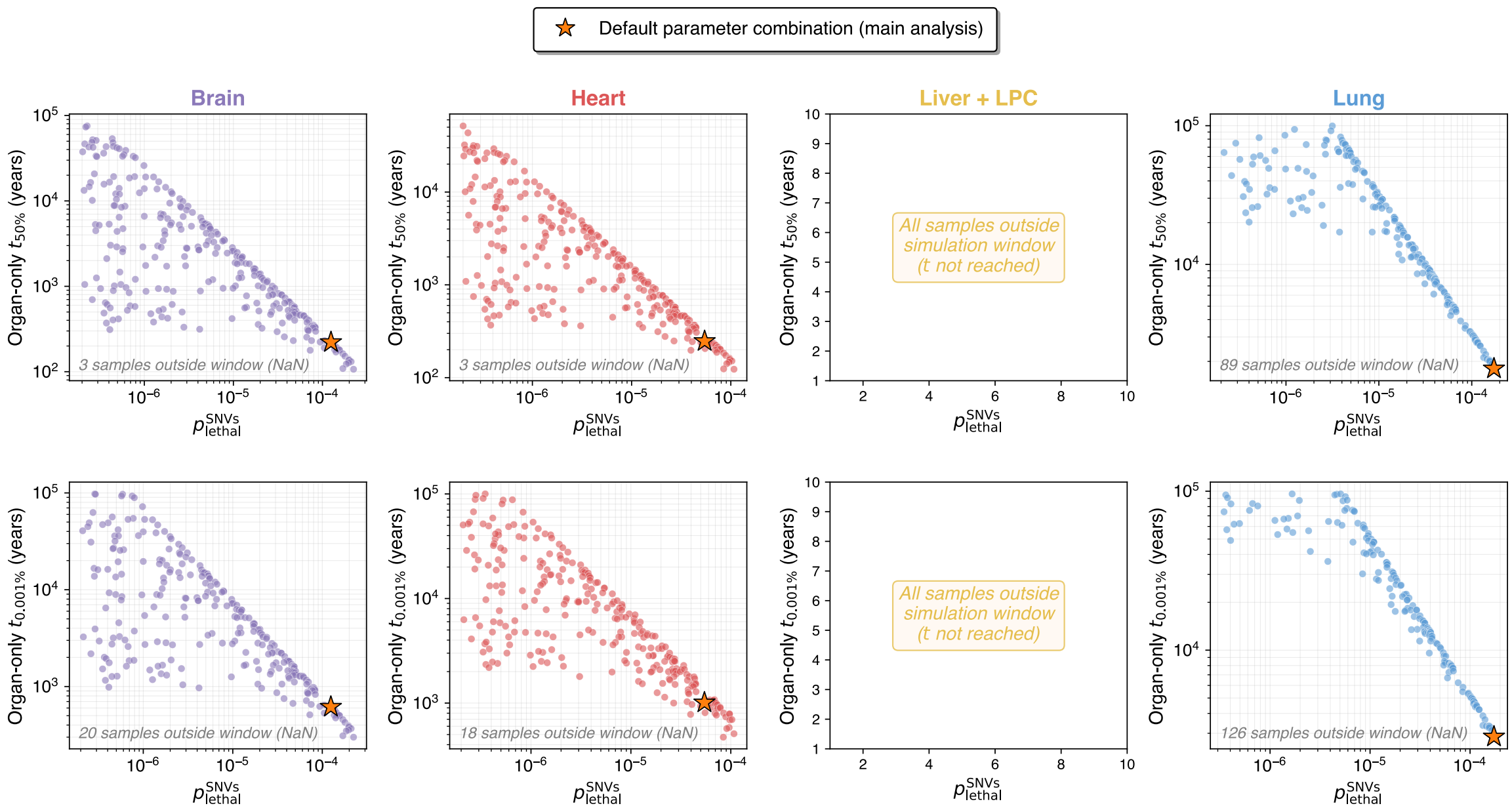

Supplementary Figure 13. Sensitivity of organ-specific predicted lifespans to uncertainty in the lethal mutation probability p_{lethal}. Each panel shows organ-only survival threshold times as a function of the per-SNV lethal probability $p_{\text{lethal}}^{\text{SNVs}}$, with $p_{\text{lethal}}^{\text{indels}}$ sampled independently and simultaneously (see Methods). Each dot represents one of $n = 300$ parameter sets drawn from a log-uniform distribution spanning the full biologically plausible range. Top row: organ-only median lifespan ($t_{50\%} = t_{\text{med}}$, the time at which 50% of the simulated organ-only population survives). Bottom row: organ-only maximum lifespan ($t_{0.001\%} = t_{\max}$, the time at which 10^{-3}% of the organ-only population survives; equivalent to 1 in 100,000 individuals). Columns: Brain (Model II), Heart (Model II), Liver + LPC (Model IIIB), and Lung (Model IIIC), from left to right. The orange star marks the default parameter set used in the main analysis. Both axes are log-scaled. The number of parameter sets for which the corresponding survival threshold was not reached within the simulation window (reported as NaN) is indicated in the lower-left corner of affected panels. Liver + LPC panels are empty because the respective organ-only survival never falls below either threshold across the entire sampled parameter space, indicating complete insensitivity of the proliferating tissue model to p_{lethal} within biologically plausible bounds.

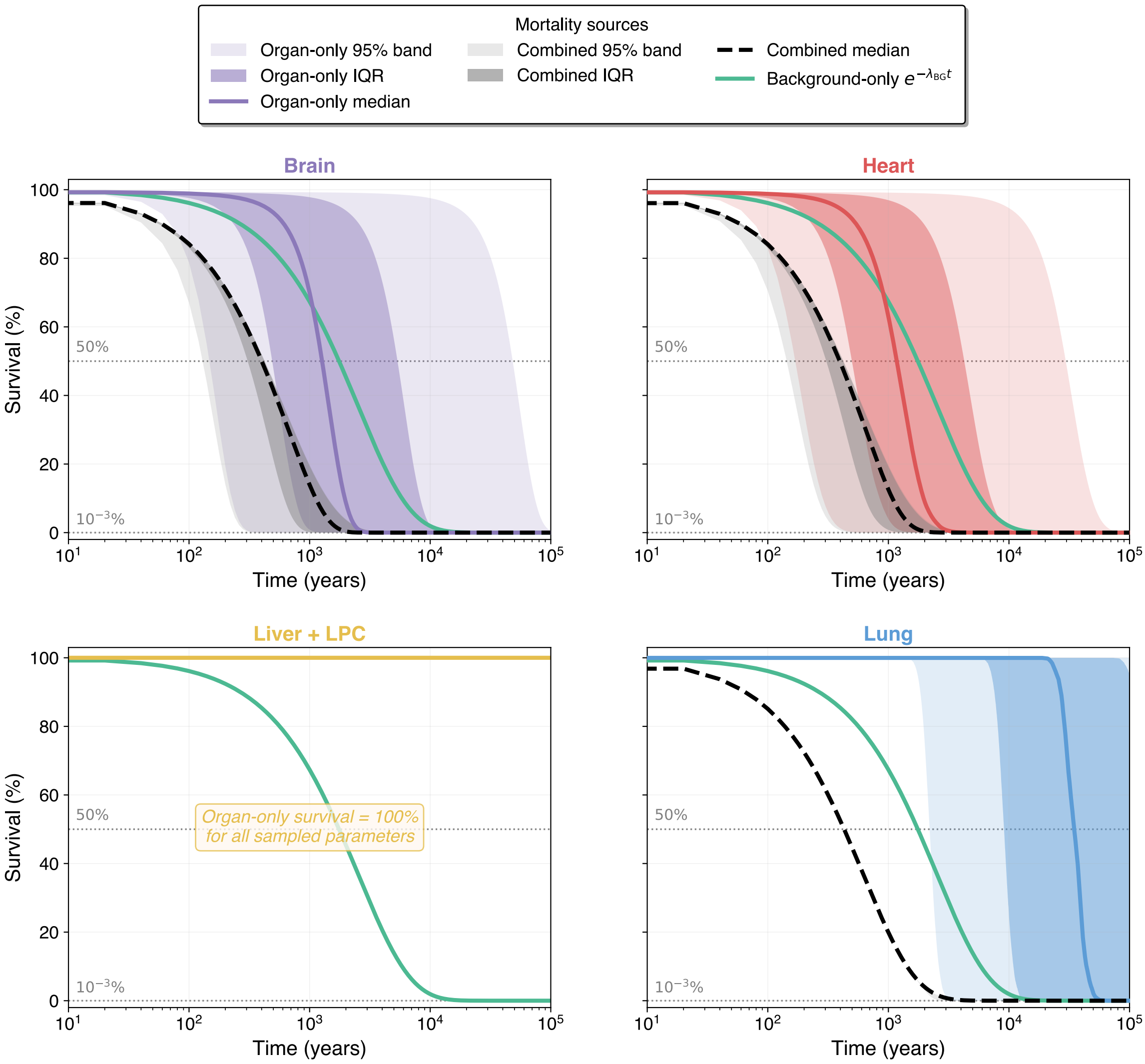

Supplementary Figure 14. Uncertainty in predicted survival curves arising from variation in p_{lethal}. Each panel shows survival as a function of time for one organ, with shaded bands reflecting the spread across $n = 300$ independently sampled p_{lethal} parameter sets. Organ-only survival (coloured bands and organ-only mean lines) isolates the mutation-driven mortality component, excluding background mortality. Combined survival (grey bands and dashed black line) multiplies organ-only survival by the deterministic background survival term $S_{\text{BG}}(t) = e^{-\lambda_{\text{BG}}t}$, where λ_{BG} is the Swiss 30-year-old hazard rate. Background survival (solid green line) is shown separately for reference. For each survival component, the darker shaded region spans the interquartile range (IQR, 25th–75th percentile) and the lighter shaded region spans the 95% uncertainty band (2.5th–97.5th percentile) across parameter sets. Solid coloured and dashed black lines show the respective medians. Horizontal dotted grey lines mark the 50% and 10^{-3}% survival thresholds used to define median and maximum lifespans throughout the manuscript. The time axis is log-scaled. Liver + LPC panel: organ-only survival equals 100% for all sampled parameter combinations, illustrating that predicted lifespan for this tissue is determined entirely by background mortality regardless of p_{lethal}.
